# Sustainable hospital accreditation in low- and middle-income countries: lessons for global stakeholders from Sri Lanka's discontinued national program

**DOI:** 10.1108/IJHCQA-04-2026-0104

**Published:** 2026-08-31

**Authors:** Dilantha Dharmagunawardene, Mark Avery, David Greenfield, Paula Bowman, Reece Hinchcliff

**Affiliations:** Department of Management, Griffith Business School, Griffith University, Queensland, Australia; Patient Safety and Accreditation Bureau, Ministry of Health and Mass Media, Colombo, Sri Lanka; Griffith University, Queensland, Australia; School of Population Health, University of New South Wales Medicine and Health, Sydney, Australia; School of Public Health and Social Work, Faculty of Health, Queensland University of Technology, Kelvin Grove, Australia

**Keywords:** Hospital accreditation programs, Quality and safety management, Qualitative case study, Low- and middle-income countries, Sri Lanka

## Abstract

**Purpose:**

Poor quality and unsafe healthcare exert an immense burden on resource-constrained health systems in Low- and Middle-Income Countries (LMICs). Hospital accreditation programs (HAPs) can help address this challenge, but many programs are not sustained, and limited research explains why. This case study aimed to inform future HAP implementation by examining the emergence, progression and discontinuation of the Sri Lankan Hospital Accreditation Program (SLHAP).

**Design/methodology/approach:**

Purposively selected accreditation stakeholders (*n* = 18), directly involved in the SLHAP, were interviewed using a validated, semi-structured guide. Data were thematically analysed, with coding validated.

**Findings:**

The SLHAP emerged through strong government leadership, with international technical and financial assistance. It progressed through standard practices, including developing standards, surveyor training and compliance gap analysis, facilitated by robust organisational structures. Discontinuation was due to limitations in project management and in the integration of HAP elements, resulting from governance constraints and leadership turnover. This resulted in frequent changes of plans, delays in adapting and communicating standards, and limited awareness and competencies among stakeholders.

**Practical implications:**

A complex interaction of myriad factors produced the SLHAP's trajectory. The primary factors found to have led to discontinuation (project management, leadership turnover and governance constraints) can be addressed prospectively in other LMICs through comprehensive, effective and long-term project planning and management.

**Originality/value:**

This study offers an in-depth exploration of meso- and micro-level factors shaping the overall trajectory of SLHAP, highlighting the importance of strengthening project management and meticulous integration of HAP elements. Therefore, SLHAP gives a unique opportunity to learn from a discontinued HAP in an LMIC.

## Plain language summary

The Sri Lankan Hospital Accreditation Program, initiated in 2015 and discontinued in 2019, provides a unique opportunity for global accreditation stakeholders to learn from the failure of a hospital accreditation program (HAP). The discontinuation was due to multiple meso- and micro-level factors, resulting in inadequate project management and limited integration and coordination of accreditation program elements.

## Introduction

Quality and safety management (QSM) in hospitals ensures that health services are delivered in a safe, timely, efficient, effective, equitable and patient-centred manner (Committee on Quality of Health Care in America, 2001). Hospital accreditation programs (HAPs) are imperative to achieve QSM systematically through maintaining and continuously improving healthcare and organisational standards (Kiran *et al.*, 2024).

HAPs involve an independent, external body evaluating an organisation's performance against predefined standards via a team of trained assessors. They are increasingly used in low- and middle-income countries (LMICs), which experience the largest global burden of poor quality and unsafe healthcare (World Health Organization, 2024). Many HAPs in LMICs have been discontinued, having consumed significant resources and attention (Dharmagunawardene *et al.*, 2025). Contributing factors include insufficient resources, non-alignment of HAPs with regulatory and governance architecture, and limited patient safety culture (Dharmagunawardene *et al.*, 2025; Mansour *et al.*, 2020).

**Figure 1:**
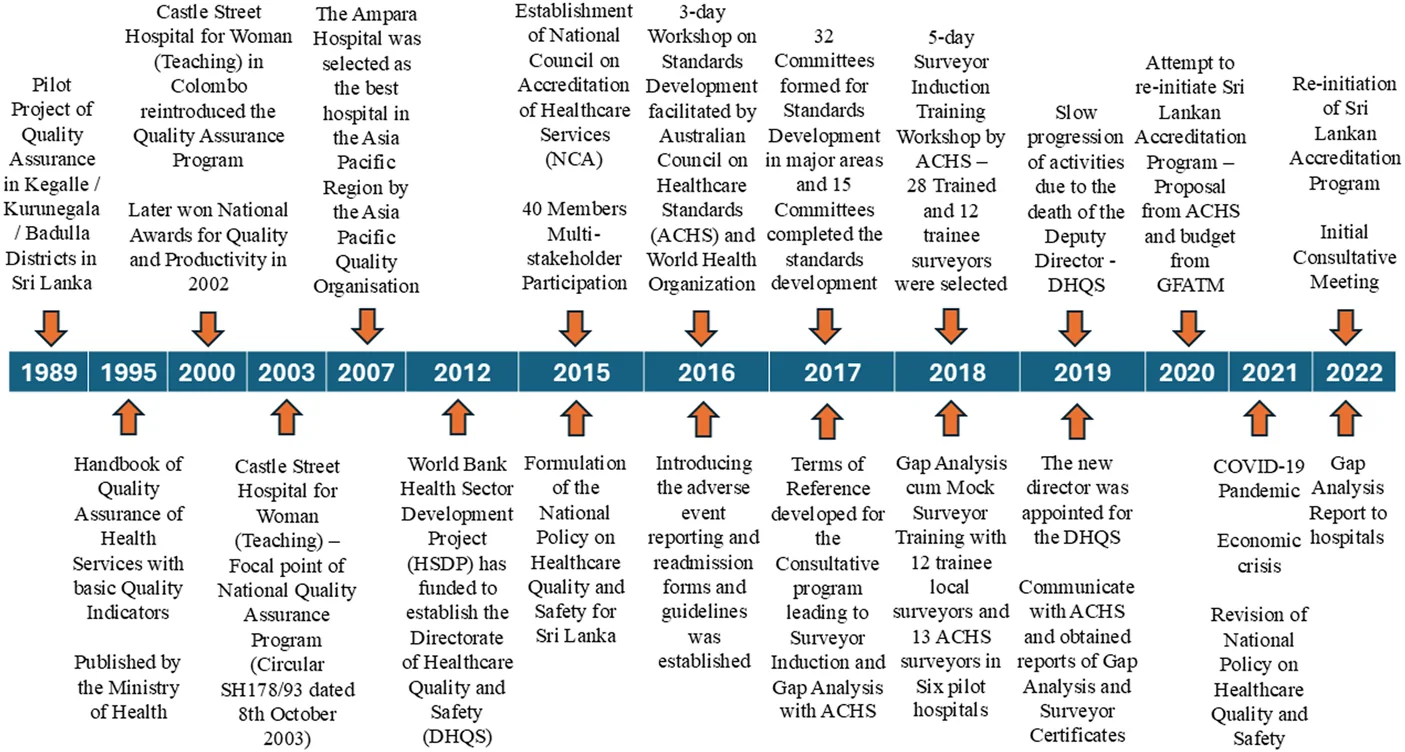
Trajectory of accreditation and quality assurance program in Sri Lanka

Research on contributors to the emergence, progression, and eventual discontinuation of HAPs in LMICs is limited. This study, focusing on the Sri Lankan Hospital Accreditation Program (SLHAP), aimed to address this gap. Most research on this topic involves description or evaluation of successful programs, rather than critical analysis of programs that are viewed as failures, making this study a unique contribution to the literature. The SLHAP was initiated in 2015 by the Directorate of Healthcare Quality and Safety (DHQS), Ministry of Health (MoH) (Directorate of Healthcare Quality and Safety, 2024). The initiative was discontinued in 2019 (Dharmagunawardene *et al.*, 2024; Karandagoda, 2023) (Figure 1). This study explores the key drivers that contributed to the emergence, progression and discontinuation of the SLHAP. Lessons from this experience are distilled as guidance for other LMICs embarking on sustainable accreditation reforms.

## Methods

An interpretivist case study was employed to capture the lived experiences of purposively selected participants (Palinkas *et al.*, 2015), who represented six groups directly involved in establishing the SLHAP. Initially, four groups were identified, and an additional two groups were recognised through snowball sampling (Table 1) (Naderifar *et al.*, 2017).

**Table 1:** Details of study participants for this research project

Stakeholder categories	Group description	Number of participants	Participant code
Sri Lankan policy officials	Sri Lankan MoH officials	03	S-03-MS
	Officials who are currently working with the DHQS *	03	S-03-MN
	Officials who previously worked at the DHQS *	03	S-03-MS
Sri Lankan health professionals	Sri Lankan Hospital administrators	03	S-03-DS
	Sri Lankan health professionals, trained by the ACHS, as surveyors	03	S-03-AS
International surveyors	Surveyors from the ACHS	03	S-03-AA

**Note(s):** * Groups identified using snowball sampling

**Figure 2:**
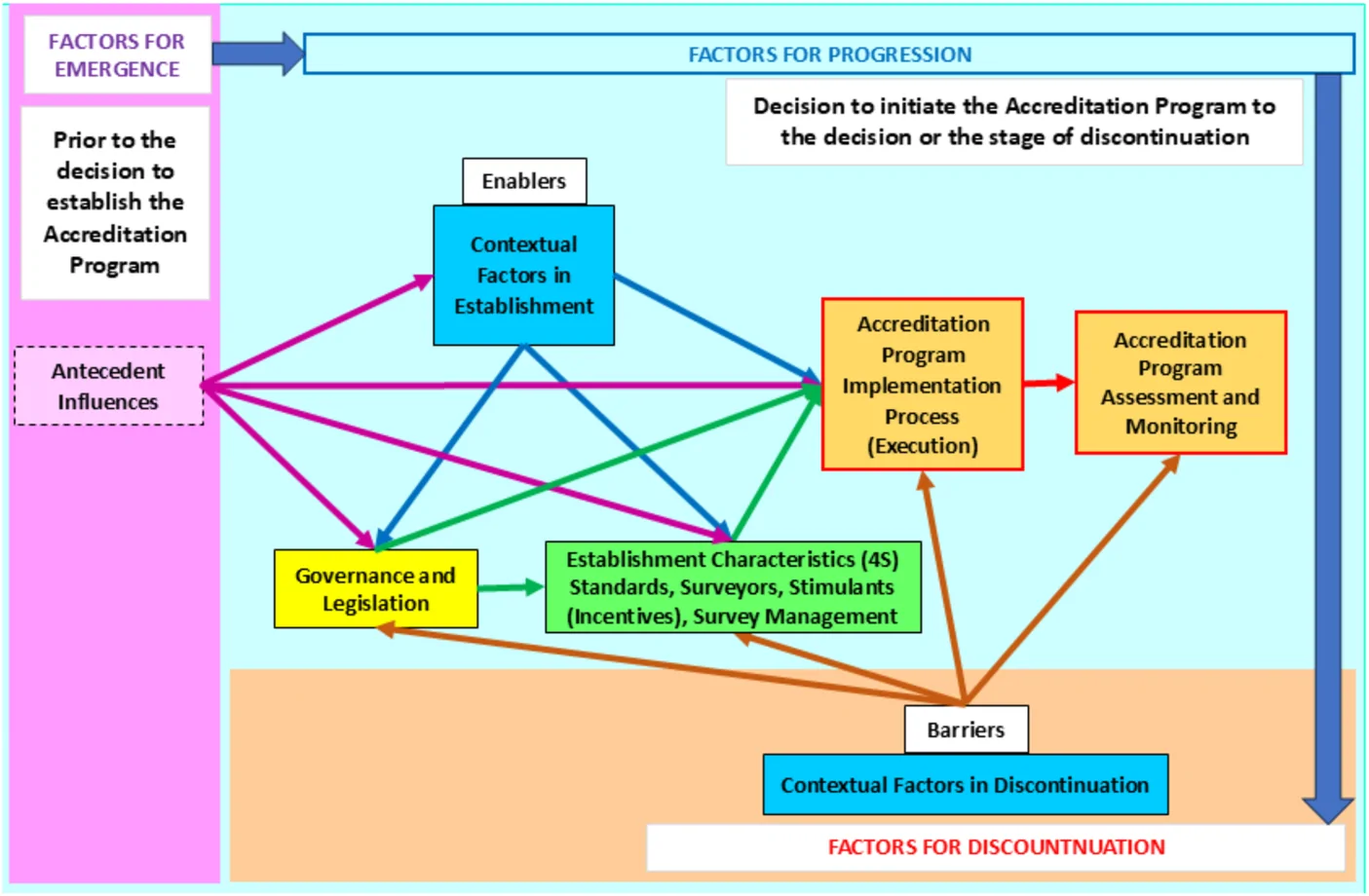
Modified ACES-GLEAM framework (Dharmagunawardene *et al.*, 2025)

The interview guide was structured using a modification of the ACES–GLEAM framework, which was developed in a published scoping review (Dharmagunawardene *et al.*, 2025). The Framework outlines core components of a HAP trajectory (Figure 2). The interview guide was checked for face validity and pilot-tested with non-participating Sri Lankan healthcare administrators. During April 2024, semi-structured interviews were conducted in English.

Snowball sampling, combined with iterative data analysis, continued through to data saturation (Saunders *et al.*, 2018). After developing a codebook (Supplementary File I), meaningful segments of text were extracted from transcripts (Saunders *et al.*, 2023) using Lumivero's NVivo qualitative data analysis software (Jackson and Bazeley, 2019). Data analysis of 10% of coded text segments was reviewed independently by team members for validation (Saunders *et al.*, 2023).

**Figure 3:**
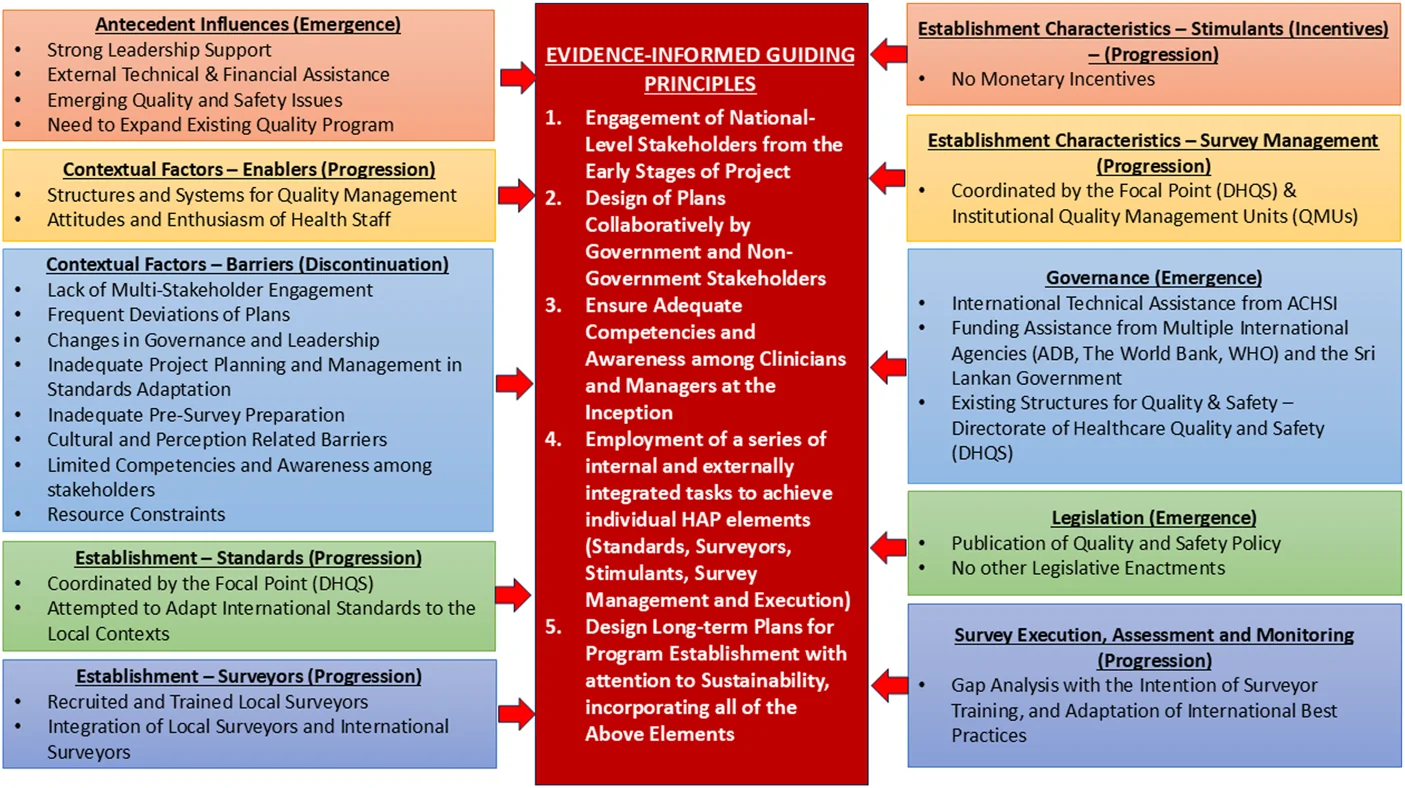
Summary of findings of the Sri Lankan case study and implications for practice, as evidence-informed guiding principles

When presenting the results, the ACES–GLEAM Framework, which guided data collection, was reorganised into three analytical categories as factors contributing to emergence, progression and discontinuation. Antecedent influences, governance and legislation domains were categorised as factors of emergence. Hindering contextual factors were classified with the factors of discontinuation. The remaining domains (enablers, standards, surveyors, stimulants, surveys and survey execution with assessment and monitoring) were broadly classified as factors contributing to the progression (Figure 3).

Ethics approval was granted by the Human Research Ethics Committee of [*recognised university, to be included after anonymised peer review*] (Ethics Approval Number 6951).

## Results

The results were categorised into the stages of emergence, progression and discontinuity of SLHAP, and the trajectory based on the expressions of the study participants is illustrated in Figure 1. According to this figure, the SLHAP was a continuation of a long-standing quality assurance program that was initiated in 1989. The SLHAP was initiated in 2015, with the establishment of the National Council on Accreditation of Healthcare Services (NCA) as the steering committee, and the DHQS as the implementation body. Context-specific standards development was initiated in 2016 with technical assistance from the Australian Council on Healthcare Standards (ACHS), which was not completed by the time of surveyor training. Then, the surveyor training was conducted in 2018 with the technical assistance of ACHS, using ACHS Equip6 standards, and 12 Sri Lankan surveyors were selected. These surveyors and ACHS surveyors conducted a gap analysis in six Sri Lankan pilot hospitals with three objectives: (1) to provide experiential training for the Sri Lankan surveyors, (2) to assess the feasibility of assessing public hospitals using ACHS standards and (3) to evaluate the existing gaps in public hospitals. Despite creating awareness and enthusiasm among a few selected stakeholders, the SLHAP was discontinued in 2019 due to a myriad of factors, without progressing to a stage of a fully-developed HAP with formally accredited hospitals or changes in accreditation governance.

The contributory factors for emergence, progression and discontinuation, which are classified according to the ACES–GLEAM framework, are illustrated in Figure 3.

### Emergence of the SLHAP

Four factors shaped the emergence of the SLHAP: leadership from MoH policy officials, the need to enhance safety and quality capabilities across the system, emerging capabilities of private health organisations, and the opportunity for international assistance.

Participants reported that the SLHAP was initiated due to the MoH policy officials' leadership. Critically, they were positioned to introduce the HAP, following their exposure to international HAPs.

The first one is probably the leading one, the personal interest and initiative, …within that and the enthusiasm of these directors … S-03-AS1

I had an opportunity to visit Singapore for a JCI [Joint Commission International] accreditation workshop in 2011 or 2012 … Also, we had the opportunity to go for assessors' training of the ACHS accreditation programme … S-03-MS3

The policy officials identified the need to uplift a long-standing hospital quality program by establishing the SLHAP. The SLHAP’s emergence was further fuelled by increasing concerns regarding medical litigation, the broader context of socio-economic transformation, and increased prosperity during the country's immediate post-civil-war period.

In 1988 and 89, the first quality improvement programme was initiated as a small pilot project by Doctor F …. S-03-MN4

… Directorate of Healthcare Quality and Safety had a policy initially. I think documented around 2010, and they mentioned some key strategies to safeguard the quality and safety to establish the accreditation system …... S-03-MS4

… there were legal processes going on during that time, some allegations, some court cases against the health ministry, against the individual doctors, which are related to medical negligence, the resources, quality of care … S-03-MS6

Sri Lanka, during that time, we were a stable country [after the civil war] … S-03-MS6

The motivation of policy officials was also influenced by their awareness of local, well-resourced private hospitals’ voluntary participation in international HAPs as a measure to promote medical tourism. The SLHAP presented a strategy to enhance the public sector’s capabilities in improving quality and safety.

… then the private hospitals, in Sri Lanka, have adopted JCI and also the ACHS. One example was JCI, which was given to two hospitals, and these hospitals were promoting accreditation not only in Sri Lanka, but also outside Sri Lanka, and they were attracting many foreigners to medical care, which brings us foreign currency. S-03-MS4

International bodies shaped the emergence of the SLHAP externally through this networking. The DHQS coordinated and collaborated with international agencies to initiate the SLHAP, including ACHS for technical assistance and the World Health Organization (WHO) for financial assistance.

… Ministry of Health in Sri Lanka made contact with ACHS through the International Division, and an agreement was reached between the Sri Lankan Ministry and the ACHS … S-03-AA3

We wrote a proposal to the WHO to visit ACHS to study the system … WHO agreed …...I met Doctor K from ACHS when he came to Sri Lanka …. … At that time, I had a detailed discussion with him in 2014 about the ACHS application process …. S-03-MS3

… the quality directorate [DHQS] was coordinating all the activities for the quality and safety … S-03-MS4

### Progression of the SLHAP

The SLHAP progressed from an idea into reality through three interrelated actions. First, a legislative and policy governance system was established to provide context for the program. At the same time, program components were developed, including accreditation standards, surveyor recruitment and training, survey management (logistics and communication) and survey execution. These mechanisms were facilitated by an accreditation-quality improvement culture consisting of staff competency and organisational culture-related enablers.

#### Governance and the legislative system

Two policy officials expressed that the National Policy on Health Care Quality and Safety, published in 2015, was a significant enabler of program development and progression. The governance mechanisms were financially facilitated through funding by international donor agencies. ACHS facilitated implementation by training senior officials and local surveyors, developing standards and conducting compliance gap analyses.

The 1st draft [Quality Policy] was prepared …, was published in 2015, and amended in 2021. S-03-MN4

… We had World Bank, we had ADB, everything … No barriers because anyway we started the process with funding in hand … So it would have been the barrier, but our planning was so careful, and we made the funding available and then started the work S-03-MS1

… in 2016, 2017, we invited ACHS again; the WHO sponsored it … on top of that, I have allocated some $250,000–300,000 for this accreditation process … S-03-MS3

#### Development of program components – standards

The DHQS engaged in a multistakeholder collaborative process to produce the SLHAP standards by adapting ACHS standards to the Sri Lankan context. DHQS policy officials reviewed three international standards to assess their local feasibility and applicability. The ACHS standards were selected for use in the SLHAP. The ACHS was consulted to educate Sri Lankan stakeholders about the accreditation program and associated processes. Following training, the ACHS standards were reviewed by local experts.

We focus mainly on the [A] document … is not really up to the expectation … Out of the [B] and the [C] documents, more palatable to us was the [C] S-03-MS6

A workshop held with quality accreditation experts …. Invited all the professional bodies, specialties, subspecialties, representatives from the colleges, and all the disciplines … S-03-AS1

We, [DHQS], actually coordinated all the activities from the development of standards for each standard domain …. We developed and conducted several meetings to finalise those to get the consensus from the expert …. S-03-MS4

#### Development of program components – surveyors: recruitment and training

Participants reported that the initial trainee surveyor cohort comprised 12 representatives of professional colleges or those involved with the DHQS. Recruiting Sri Lankan health staff from diverse specialities empowered local change agents and diffused knowledge and capabilities across the health system. ACHS surveyors were engaged in the initial induction and subsequent experiential training (during gap analysis). Surveyors and MoH officials viewed training as comprehensive and interactive, enabling the acquisition of required competencies to conduct valid, credible assessments.

The surveyor recruitment process was done by the ministry's high officials; it's like the steering committee members were involved for that … S-03-MS4

They [Ministry] actually use their internal assessors, which was the intention, to transfer some of the standards and assessors back to Sri Lanka …. S-03-AA1

Training was done in-house as a five-day workshop with the international trainers from ACHS …. It was complemented by the secondary gap analysis with the support from the ACHS assessors … S-03-AS3

#### Development of program components – surveys: management and execution

Participants stated that the SLHAP's progression was grounded in effective survey management activities (communication, coordination and logistics arrangements) with the six public hospitals. These hospitals were selected for gap analysis (survey execution) as a pilot project. Activities were coordinated by DHQS at the national level and by the hospital quality management units (QMUs).

It was managed from the quality and safety directorate … the logistics and communication were done appropriately, and there were no gaps S-03-AS2

It was conducted through our hospital quality and safety unit … There is an appointed medical officer to conduct these quality and safety activities …. S-03-DS2

A survey execution, in the form of a standards compliance gap analysis, was conducted to assess the feasibility and applicability of ACHS standards in the Sri Lankan context. The task had the added benefits of further training for surveyors. Surveys were conducted by both Sri Lankan and ACHS surveyors, with stakeholders reporting that the involvement of ACHS surveyors promoted confidence, reliability of assessment and effective communication.

… we were to test the application of contemporary developed countries' standards for safety and quality. S-03-AA3

… because it was done as a mock audit to train assessors and to see the feasibility of how we can do the audit process S-03-AS1

### Progression of an accreditation – quality improvement culture: staff competency and organisational culture-related enablers

Participants perceived that the mix of local and international surveyors promoted knowledge sharing, attention to local context and credibility. The combination of surveyors enhanced a culture of safety and quality through improved health staff competencies and developed a sustainable, competent local surveyor workforce.

… it's like a training for our surveyors as well … …. I think it was, complemented by the gap analysis we perform with the support not just from the trainers, but from the international assessors who came for the gap analysis S-03-MS4

… firstly, I think that a louder transfer of skills and knowledge to the staff at the hospital … S-03-AS1

Almost all participants, particularly ACHS surveyors, commended health staff for their enthusiasm, commitment, keenness to learn and engagement with the quality improvement process. Participants commended the interest, commitment, support and collaboration of the leaders of the MoH, DHQS and hospital administrators. These organisational culture aspects, with local capacity development, contributed to the initial successful progression.

… they were also keen to hear the feedback from us in terms of things they could do better … S-03-AA1

One thing that always impressed me in Sri Lanka was the enthusiasm … S-03-AA2

There was very strong engagement everywhere we went S-03-AA3

### Discontinuation of the SLHAP

The discontinuation of SLHAP resulted from the combination of barriers linked to governance and leadership, HAP components, contextual, personnel and cultural issues. They were related to resources, skills and competencies, as well as organisational culture-related barriers.

#### Constraints in governance and leadership

Stakeholders reported that governance and leadership-related constraints were a major impediment to SLHAP. This included a lack of shared decision-making, limited multi-stakeholder involvement, poor communication between the MoH and ACHS, and regular deviation from previously agreed-upon project management plans, contributing to discontinuation.

We were not able to incorporate top and bottom or other layers of the health care providers and even the policy makers into this. S-03-MS6

… there was a big communication gap within our higher officials and also with the accreditation authorities S-03-MS4

… you had to align with their [higher officials] agenda and their guidance. So, they need some amendments to get a deviation from the plan … S-03-MS4

Leadership-related problems, such as frequent changes, including the unexpected death of the Director of Quality, have reduced institutional memory. The lack of information and knowledge transfer during turnover resulted in a decline in understanding and process competencies. As a result, inconsistencies and ambiguities arose in the overall guidance.

One person is getting trained to do that, and he is transferring to another place … So, we have to train another person for that particular job … So that's one aspect that is also one reason for not sustaining this patient safety quality programme. S-03-MN3

A lot of things were done by the previous director, and thereafter, there was no continuation … Then, a director was there for one year. Then I assumed duty. So, what he has done earlier in the ACHS accreditation, there is no evidence, there is no institutional memory there. There are no records as such S-03-MS2

#### Failure to adapt the standards to the local context

Participants reported that while adapting ACHS standards to the local context was agreed upon, no adaptation ultimately occurred. This was due to the later decision of MoH policy officials, who independently considered ACHS standards appropriate. ACHS and local surveyors held concerns about standards not aligning with local system constraints and context. Stakeholders noted that the version of the ACHS standards underwent a change due to the lengthy project period, rendering the original training and initial gap analysis less relevant. The failure to adapt the standards was viewed as a major reason for discontinuation, representing a lack of multi-stakeholder engagement and shared decision-making.

… the committee meetings probably had few and not continued further … developing a fully established criteria and guidelines to suit the Sri Lankan setup was slowly fading away halfway through … S-03-AS1

… we actually put great effort into developing those standards, which are country-specific. But at last, it was not considered for the training … And at least we have to do a pilot before that. No, it was not planned at that time. S-03-MS4

Sri Lanka is not to Australia, and how to establish our cultural differences, socio-cultural differences, we have to address that context S-03-AS1

… the participants were only doctors, professional bodies, colleges, representatives from the professional colleges and directors, and had no other stakeholders in those committees, and again, I think, a failure. S-03-MS6

When I was going through this process [standards adaptation], what I felt was that it was a bit of a difficult task. So, as they are from the Australian standards, they have even considered all minor points. Whereas in Sri Lanka, we have not achieved, we have not gone through up to that level. So, whereas in these lower-middle-income countries, it is a bit difficult all of a sudden to adhere to even minor things, such as following standards …. And later, maybe in a later phase, we can think of developed country accreditation standards. Otherwise, it is very difficult to implement this …. So that is why I am saying it is difficult to adopt somebody else's one to the Sri Lankan setting. S-03-MS5

#### Constraints in surveyor recruitment and engagement

Despite a strong belief in the quality of surveyor training, stakeholders reported concerns about surveyor recruitment. These concerns stemmed from not having or adhering to specific recruitment criteria, limited representation of all professional categories, a lack of preparation or awareness about accreditation prior to surveyor recruitment and poor engagement with MoH activities. Poorly planned recruitment and subsequent limited engagement resulted in a lack of collaboration between the MoH and trained surveyors.

We have to select those surveyors, adhering to the protocol. But it did not happen … S-03-MS4

I [one of the trained surveyors] was not aware of it …. …. Why are they asking me to join? What could be my contribution? So that initial preparation was not there …. S-03-AS3

If you go to the Ministry of Health and request a list of successful assessors from that programme, it is not available … they do not know … S-03-AS1

… they [local surveyors] are not giving anything to the Ministry of Health. S-03-MS2

#### Constraints in survey management

Sri Lankan surveyors and hospital administrators reported inadequate pre-survey communication strategies, resulting in minimal awareness among health staff about the standards and assessment processes, as well as the purpose of the gap analysis. Inadequate preparation of hospitals due to a lack of time before program implementation was highlighted by participants outside the MoH. Similarly, despite DHQS officials’ mention of training programs for hospital staff, only one hospital administrator reported receiving any training. One senior MoH officer indicated that they wanted to assess the situation during the gap analysis without prior information given to the hospitals. Overall, these differences in statements between participants revealed no standard process of communicating standards to the pilot settings of gap analysis.

After the survey, and even before the survey, nothing happened, nothing happened. And after the survey, nothing was said about the survey. S-03-DS1

… the institutions were not properly informed and not properly prepared for that …. S-03-AS3

#### Constraints in survey execution

The ACHS and local surveyors mentioned multiple barriers to survey execution, such as contextual mismatches, resource limitations within hospitals and problems in the hospitals (overcrowding and ongoing construction work), to implement standards.

Some of the infection control stuff was a challenge, but it was what people had to live with. There weren't enough beds … S-03-AA1

I think it was harder on the first day of the first hospital because the culture shock of such a different system from our own was at its highest … S-03-AA3

Hospital administrators expressed dissatisfaction with the HAP because they received no feedback or a final report. MoH policy officials received a final report almost one year after completing the surveys, but dissemination was limited to top-level policy officials. Nevertheless, most participants outside the MoH were unaware of the program assessment, monitoring or evaluation, or the availability of the gap analysis reports. This inadequate feedback led to limited learning and the disengagement of frontline stakeholders.

I was searching for a report … So many years later, only I got that report …. It was not given S-03-DS3

#### Program resource-related barriers

Program resource constraints, such as inadequate resources, lack of equipment and poor infrastructure, were highlighted by participants, except for higher MoH policy officials. The main reported financial barriers were the high cost of accreditation, the need for costly investments for related institutional changes and the lack of prioritisation of allocations amidst other demands. The rapid turnover of staff involved in quality and safety activities was the primary reported human resource-related barrier.

… even infrastructure facilities … minimum number of personnel and equipment requirement ….so is the availability of trained nursing officers, other supporting staff. So, it did not have all the wheels as per the requirements …. S-03-AS1

… the seed funding for that exercise came from the World Bank, which was not a sustainable thing S-03-AA3

They focus rather on the day-to-day availability of paracetamol in the hospital, which was the most important thing for them, rather than quality improvements … S-03-AS1

#### Competency-related barriers

Stakeholders reported that staff turnover led to competency-related constraints. Inadequate accreditation skills and knowledge, and quality and safety practices, existed among middle-level managers. Front-line hospital staff's lack of education, training and awareness on accreditation-related topics, in addition to a lack of expertise at the DHQS, were reported by participants. As a result, middle-level managers were unable to ensure staff engagement and support for accreditation, which contributed to its discontinuation.

… There was actually no specialised specialist at that time, either an administrator or a community physician in that unit [DHQS] …. S-03-MS4

… Most of the other heads of the institutions didn't know about this because they have not given any training, no more awareness on this accreditation … S-03-DS1

I didn't feel like there was good knowledge before we arrived. I didn't feel like the hospitals knew the standards before we got there … S-03-AA3

… We were not able to create awareness among our stakeholders and give them the active support … S-03-MS6

#### Organisational culture-related barriers

Participants stated there was a broad group of organisational culture- or perception-related barriers, which included negative perceptions towards accreditation, poor perceptions of multi-stakeholder engagement and negative perceptions of teamwork. Poor quality culture-related perceptions were reported, such as “afraid of audits”, “fault-finding missions” and “assessments lead to punishment”.

… but even from the top to bottom, from the politicians to our local hospital administrator concentrating minimally, they are not giving that priority … We are not yet ready psychologically, physically, individually and institutional basis. S-03-AS2

… That was not in the mainstream of the ministry. It was not in the ministry agenda …. It is not a common initiative. S-03-MS6

Sometimes, our mindset, people think it is like auditing. People with that name behind, they think it is punishment like finding faults … S-03-AS1

They don't realise the benefits of accreditation to the patient and to the health staff S-03-MN3

Negative perceptions among clinicians and policymakers included less support for quality, safety and accreditation-related activities, and more prioritisation of clinical work volume. Other identified cultural barriers to sustainability among leaders included: unnecessary influences from the hierarchy, working based on personal agendas, not involving all leaders in the hierarchy and trying to pursue accreditation as an isolated initiative.

Policy makers, I don't think they have ever paid attention to the accreditation … It was very difficult to get down clinicians to discuss these things … they are very reluctant. S-03-MS6

… from the ministry side, sometimes we have to adhere to the influences from higher officials S-03-MS4

## Discussion

This study explored key drivers that contributed to the emergence, progression, and discontinuation of the SLHAP. The study’s strength lies in its critical examination of a failed (rather than successful) HAP, as well as the breadth of participants from diverse backgrounds directly involved in the SLHAP, which elicits rich and in-depth information that can guide the accreditation activities of stakeholders in other LMICs.

The limited prior research on this topic has attributed the failure of HAPs in LMICs to resource scarcity and weak pre-existing infrastructure (Bukonda *et al.*, 2003; Dharmagunawardene *et al.*, 2025; Mansour *et al.*, 2020, 2021). Conversely, this case study provides a counterexample, making a novel contribution to the literature. Despite having pre-existing quality and safety structures, donor funding and technical support, the Sri Lankan HAP still failed to be sustained. This case study revealed that the main factors impeding implementation were related to project management and governance dynamics, including leadership turnover, inadequate adaptation of standards and limited feedback, which reduced stakeholder engagement. These factors may be present in other examples of failed HAPs in LMICs, but have remained invisible within the existing literature. This paper has distilled these unique findings and translated them into a set of concrete, transferable HAP design principles for donor agencies, ministries of health and accreditation bodies to maximise the chances of implementation success and sustainability.

Similar to the SLHAP, leadership turnovers are frequently highlighted as contributory factors for the discontinuation of HAPs in LMICs (Dharmagunawardene *et al.*, 2025; Mansour *et al.*, 2021). In the SLHAP, this was further exacerbated by the non-transfer of institutional memory. A recent systematic review examining sustainability in 124 healthcare improvement programs highlighted that the key barriers to longevity were leadership and related support, training and supervision, consistent staffing and low turnover (Zurynski *et al.*, 2023).

Leadership and governance dynamics in SLHAP led to delays in adapting and communicating standards. This has led to limited competency and awareness of accreditation among health staff, resulting in minimal staff engagement. Staff competencies were found to be an important barrier to sustainability because they impact implementation through facilitation of continuous quality improvement and engagement (Alhawajreh *et al.*, 2023). Well-established staff training programs are a feature of ongoing accreditation programs in Jordan and SafeCare (Arabji, 2013; Spieker, 2020). Staff competencies and education are noted as foundational pillars for organisational patient safety programs (International Society for Quality in Health Care, 2025) and are also imperative for enhancing staff engagement. Accordingly, staff engagement is another critical factor for the sustainability of HAPs (Greenfield *et al.*, 2011; Hinchcliff *et al.*, 2013a; Hussein *et al.*, 2025).

Delays in adaptation and communication standards, and limited staff awareness, competencies and engagement, were all due to the sudden and unplanned changes experienced by the SLHAP. These changes were largely a result of project management constraints in scope, time, quality, communication, stakeholders and human resources (Freire *et al.*, 2016; PMI - Project Management Institute, 2012), which have not been highlighted in contemporary accreditation literature or existing guidelines. Existing guidelines from the International Society for Quality in Health Care (ISQua), the WHO, and other organisations focus on the technical aspects of program development without considering programs as long-term, complex projects (International Society for Quality in Health Care, 2018; World Health Organization, 2022). Nevertheless, program complexity, inadequate planning and group design were noted in the discontinuation of healthcare improvement programs (Zurynski *et al.*, 2023).

As a further point of contrast, financial and human resources and previously weakened structures and systems were not reported as prominent barriers for the discontinuation of SLHAP, similar to other LMIC programs (Bukonda *et al.*, 2003; Dharmagunawardene *et al.*, 2025; Mansour *et al.*, 2020, 2021). Despite SLHAP discontinuation, some HAPs in LMICs have sustained operations and subsequently obtained ISQua recognition (Arabji, 2013; David and Valas, 2017; Spieker, 2020). The continuation of these HAPs has been reportedly due to sustained positive driving forces, such as leadership support, robust standards development adapted to the local context, ensuring competencies of health staff on accreditation, reliable surveyor training programs (Dharmagunawardene *et al.*, 2025; Mansour *et al.*, 2020), supportive safety and quality policies and strategies (Alhawajreh *et al.*, 2023; Hinchcliff *et al.*, 2013b; Mansour *et al.*, 2020), and incentives mainly linked to insurance systems (Mansour *et al.*, 2021).

The study revealed and confirmed in the literature that emergence, progression, and (dis)continuation result from a complex interplay of multiple factors. This result reflects the complex dynamic system of the health sector, regardless of country setting, and the challenge of achieving positive, lasting change in safety and quality dimensions (Zurynski *et al.*, 2023).

### Implications for practice

Continuation or discontinuation of HAPs are unique trajectories specific to each context but shaped by common features: leadership, communication, broad engagement of health professionals, education and training, health system and organisational resilience, and safety and quality cultures. A set of evidence-informed guidelines aligned with the key results is illustrated in Figure 3.

Effective and consistent project management significantly contributes to the implementation and outcomes of HAP. Program sustainability, including institutional memory, handover protocols for leaders, predetermined program and standard updates, and embedded medium-to long-term program evaluations, needs to be built into the initial design. The focus of internal stakeholders should be on formulating a long-term plan for establishing HAPs, incorporating the aforementioned strategies.

Formulating long-term plans should target the achievement of individual sub-components in a gradual and stepwise manner, including training for health staff and leaders, surveyor training and standards development. Similarly, external stakeholders should focus on facilitating the above individual sub-components technically and financially as specific entities, in an overall continuum of the HAP establishment pathway.

Finally, education and awareness of staff and leaders on accreditation concepts need to be conducted as a key engagement endeavour. Out of this cohort, local champions and surveyors can be recruited to advocate for, and participate in, the implementation of HAPs.

There are three main study limitations: (1) the study focused on one HAP in one country, (2) recall bias among participants and (3) participants were stakeholders involved in SLHAP, who may be biased and conceal contributing factors. However, including participants from diverse hierarchical levels and stakeholder groups minimised this risk.

## Conclusion

Understanding key drivers contributing to the emergence, progression and discontinuation of HAPs in LMICs can help improve safety and quality processes and outcomes. Success and failure result from a complex interplay of multiple factors, each countering or reinforcing each other, in a dynamic process unique to a specific setting. SLHAP provides a blend of unique and common factors that is imperative for the success and sustainability of HAPs in LMICs. Despite a certain degree of resource constraints similar to those of other LMICs, Sri Lanka had a relatively good health infrastructure and financial resources for accreditation. The discontinuation of SLHAP was uniquely and prominently linked with the issues of project management and planning, leading to incoordination between HAP elements during establishment, and this factor was limitedly explored in previous literature. Moreover, similar to other LMICs, the discontinuation of SLHAP was linked with leadership turnover, poor engagement and limited competencies of stakeholders.

Therefore, as illustrated in Figure 3, sustained leadership, coordinated and continuous local multi-stakeholder engagement and long-term planning are key to success. Additionally, a series of internal and externally integrated tasks is required to achieve individual HAP elements, recognising that the establishment of HAPs is a highly complex intervention. These aforementioned elements should be integrated into an effective project management trajectory to maximise the chances of implementation success and sustainability of HAPs.

## Supplementary Material

Data supplement 1

## Notes

The supplementary material for this article can be found online.
