# Supplementary material for: Sustainable hospital accreditation in low- and middle-income countries: lessons for global stakeholders from Sri Lanka's discontinued national program
Source: Int J Health Care Qual Assur. 2026 Aug 31:1–15. doi: 10.1108/IJHCQA-04-2026-0104 (PMC13521027; doi:10.1108/IJHCQA-04-2026-0104)
Supplement: Supplementary file 1 [file ijhcqa-04-2026-0104_suppl1.docx]

Supplementary File I – Description of Themes of ACES-GLEAM Framework and the Code Book for Thematic Analysis

| Name | Description |
| --- | --- |
| Antecedent Influences | any event or environmental factor that occurs before the establishment and has the power to have an effect on the establishment of an accreditation program. |
| Antecedent influences of the present system | Any event or environmental factor that has the power to have an effect on the failure or the success of the present system (within the public / government health sector) was included in this theme |
| Influencing Parallel External Programs | Any event or environmental factor that has the power to have an effect on the failure or the success of the present or Past system (outside the public / government health sector) was included in this theme |
| Assessment and Monitoring | Assessment of the degree of success in meeting the goals and expected results of an accreditation program; Process - series of interrelated activities and communications which accomplish services; Results - The results of the assessment of the degree of success |
| Contextual Factors | Circumstances, characteristics, and factors that contributed during the establishment phase of an accreditation program |
| Barriers | A set of circumstances and characteristics or unique factors that impede and challenge the establishment of an accreditation |
| Barrier - Establishment & Implementation Characteristics Related | Standards, Incentives, Surveyor and Survey related |
| Barrier - Governance, Survey Mx & Execution | The collection of interacting elements (institutions, people, resources) that operate and control the establishment of accreditation programs and accountability mechanisms, Survey and Survey Management Process - Survey - external peer review which measures the performance of the organisation against an agreed set of standards; Survey Management - Contracting with the client organization, planning and conducting the surveys, report writing, and performance indicators |
| Barrier - Standards | A desired and achievable level of performance against which actual performance is measured |
| Barrier - Stimulants (Incentives) | Strategies that are useful to promote and sustain accreditation programs, especially when the program is not mandatory |
| Barrier - Surveyors | Surveyor - External peer reviewer, assessor of organisational performance against agreed standards; |
| Barrier - External Factors | Factors outside the Health Sector |
| Barrier - External Support Related | Technical and Financial facilitation received from outside the accreditation system or program, including foreign entities (Support from Ministries of Health will be considered as internal, and outside to the MoH will be external) |
| Barrier - Resource-related | Human, Financial, Materials, Machines, or infrastructure (including perceptions of health staff) |
| Barrier - Financial Resources | The funds and assets that finance the accreditation program establishment and implementation |
| Barrier - Human Resources | The group of people who make up the workforce that involved in the establishment and implementation of accreditation program and their characteristics (e.g.: skills, abilities, perceptions, attitudes, values etc.) |
| Barrier - Culture-related | Common beliefs, expectations, assumptions, standards, and values shared by members of an organization. (Chalmers R, Brannan GD. Organizational Culture. [Updated 2023 May 22]. In: StatPearls [Internet]. Treasure Island (FL): StatPearls Publishing; 2024 Jan-. Available from: https://www.ncbi.nlm.nih.gov/books/NBK560543/) |
| Barrier - Education, Training and Awareness | Processes of informing, teaching and educating individuals about competencies (specific set of knowledge, skills and attitudes) in relation to accreditation establishment and implementation |
| Barrier - Leadership | Actions and people that are leading a group of people or an organization, involved in establishing and implementing accreditation program and their related characteristics |
| Barrier - System-related | System: A collection of interacting elements comprising all the organizations, institutions, and resources that are devoted to producing health actions; Governmental or Ministerial or Institutional related processes, strategies, or activities (Excluding Resources) |
| Enablers | A set of circumstances and characteristics or unique factors that support and facilitate the establishment of an accreditation |
| Enabler - Establishment & Implementation Characteristics Related | Standards, Incentives, Surveyor and Survey related |
| Enabler - Governance, Survey Mx & Execution | The collection of interacting elements (institutions, people, resources) that operate and control the establishment of accreditation programs and accountability mechanisms, Survey and Survey Management Process - Survey - external peer review which measures the performance of the organisation against an agreed set of standards; Survey Management - Contracting with the client organization, planning and conducting the surveys, report writing, and performance indicators. |
| Enabler - Standards | A desired and achievable level of performance against which actual performance is measured |
| Enabler - Stimulants (Incentives) | Strategies that are useful to promote and sustain accreditation programs, especially when the program is not mandatory |
| Enabler - Surveyors | Surveyor - External peer reviewer, assessor of organisational performance against agreed standards; |
| Enabler - External Support Related | Technical and Financial facilitation received from outside the accreditation system or program, including foreign entities (Support from Ministries of Health will be considered as internal, and outside to the MoH will be external) |
| Enabler - Resource-related | Human, Financial, Materials, Machines, or infrastructure (including perceptions of health staff) |
| Enabler - Human Resources | The group of people who make up the workforce that involved in the establishment and implementation of accreditation program and their characteristics (e.g.: skills, abilities, perceptions, attitudes, values etc.) |
| Enabler - Culture-related | Common beliefs, expectations, assumptions, standards, and values shared by members of an organization. (Chalmers R, Brannan GD. Organizational Culture. [Updated 2023 May 22]. In: StatPearls [Internet]. Treasure Island (FL): StatPearls Publishing; 2024 Jan-. Available from: https://www.ncbi.nlm.nih.gov/books/NBK560543/) |
| Enabler - Education, Training, and Awareness | Processes of informing, teaching and educating individuals about competencies (specific set of knowledge, skills and attitudes) in relation to accreditation establishment and implementation |
| Enabler - Leadership | Actions and people that are leading a group of people or an organization, involved in establishing and implementing accreditation program and their related characteristics |
| Enabler - System-related | System: A collection of interacting elements comprising all the organizations, institutions, and resources that are devoted to producing health actions; Governmental or Ministerial or Institutional related processes, strategies, or activities (Excluding Resources) |
| Establishment Characteristics Related | Standards, Incentives, Surveyor and Survey related |
| Standards | A desired and achievable level of performance against which actual performance is measured |
| Stimulants (Incentives) | Strategies that are useful to promote and sustain accreditation programs, especially when the program is not mandatory |
| Survey Management | Survey and Survey Management Process - Survey - external peer review which measures the performance of the organisation against an agreed set of standards; Survey Management - Contracting with the client organization, planning and conducting the surveys, report writing, and performance indicators |
| Surveyors | Surveyor - External peer reviewer, assessor of organisational performance against agreed standards; |
| Execution (Accreditation Program Implementation) | External peer review which measures the performance of the organisation against an agreed set of standards; Pre-survey - Any activities that were conducted prior to the external peer review ; Survey Process - series of interrelated activities and communications which accomplish services; Awarding of accreditation - series of interrelated activities and communications which accomplish services |
| Governance | The collection of interacting elements (institutions, people, resources) that operate and control the establishment of accreditation programs and accountability mechanisms (responsibility and requirement to answer for allocation, supervision, and coordination of tasks or activities) contributed to the establishment until the culmination of accreditation programs |
| Legislation | Any laws, ordinances, regulations, and policies related to the establishment of accreditation programs |
